# Effects of wound dressings containing silver on skin and immune cells

**DOI:** 10.1038/s41598-020-72249-3

**Published:** 2020-09-16

**Authors:** Kristina Nešporová, Vojtěch Pavlík, Barbora Šafránková, Hana Vágnerová, Pavel Odráška, Ondřej Žídek, Natálie Císařová, Svitlana Skoroplyas, Lukáš Kubala, Vladimír Velebný

**Affiliations:** 1 Contipro a.s., Dolni Dobrouc 401, 56102 Dolni Dobrouc, Czech Republic; 2 Third Faculty of Medicine, Charles University, Prague, Czech Republic; 3 Faculty of Natural Sciences, Charles University, Prague, Czech Republic; 4 Department of Biophysics of Immune System, Institute of Biophysics of the Czech Academy of Sciences, Brno, Czech Republic; 5 Department of Experimental Biology, Faculty of Science, Masaryk University, Brno, Czech Republic; 6 International Clinical Research Center, St. Anne’s University Hospital, Brno, Czech Republic

**Keywords:** Antimicrobials, Skin diseases, Infection, Adverse effects

## Abstract

Wound dressings with silver have been shown to be cytotoxic in vitro. However, the extrapolation of this cytotoxicity to clinical settings is unclear. We applied dressings with various forms of silver on porcine skin ex vivo and investigated silver penetration and DNA damage. We assessed antimicrobial efficacy, cytotoxicity to skin cells, and immune response induced by the dressings. All dressings elevated the DNA damage marker γ-H_2_AX and the expression of stress-related genes in explanted skin relative to control. This corresponded with the amount of silver in the skin. The dressings reduced viability, induced oxidative stress and DNA damage in skin cells, and induced the production of pro-inflammatory IL-6 by monocytes. The oxidative burst and viability of activated neutrophils decreased. The amount of silver released into the culture medium varied among the dressings and correlated with in vitro toxicity. However, antimicrobial efficiencies did not correlate strongly with the amount of silver released from the dressings. Antimicrobial efficiency and toxicity are driven by the form of silver and the construction of dressings and not only by the silver concentration. The damaging effects of silver dressings in ex vivo skin highlight the importance of thorough in vivo investigation of silver dressing toxicity.

## Introduction

Non-healing wounds present a significant burden to both patients and healthcare systems. Wound infection is a complication for both acute and surgical wounds, and bacterial colonisation and biofilm formation are present in the majority of non-healing wounds. Therefore, successful wound healing often demands antimicrobial therapy. The high prevalence of infection together with the increasing risk of antibacterial resistance is the reason why potent alternatives to antibiotics are sought.

Silver, known for its antibacterial properties for centuries, is commonly utilised in various medical devices including wound dressings. Commonly used silver salts contain ionic forms of silver (Ag^+^, Ag^2+^, Ag^3+^), while silver nanoparticles (AgNP) consist mainly of atomic silver (Ag^0^). To exert its biological activity, Ag^0^ must oxidise to silver oxide or Ag^+^. The oxidation of Ag^0^ is strongly influenced by the chemical composition of biological media and lighting conditions^1^.

The mode of action of silver in pathogen elimination is only partially understood, and multiple mechanisms are probably involved, including direct interactions between silver and bacterial cell membranes, DNA, and enzymes and proteins, and an indirect effect through the formation of reactive oxygen species (ROS)^2^. The antimicrobial activity of silver-containing devices depends on their composition (additional sorbents, biologically active compounds, biomolecules or (bio)polymers) and the surrounding environment driving the release of Ag^+^ ions.

Silver toxicity is broad-spectral and both prokaryotes and eukaryotes are affected^3^. Due to silver’s narrow therapeutic window, eukaryotic cells can be damaged by silver in the wound together with the pathogens by both direct contact with Ag^+^ or Ag^0^ nanoparticles and indirectly by ROS, which may result in suboptimal healing dynamics and outcomes. The known mechanisms of silver toxicity and cytotoxicity in vitro were summarized recently^4–7^. These included (a) the generation of ROS and the build-up of oxidative stress, (b) the disruption of mitochondrial activity, (c) the association of Ag^+^ with membranes and the production of peroxylipids, (d) the unfolding or misfolding of proteins leading to endoplasmic reticulum stress, (e) apoptosis and autophagy, (f) the activation of redox-sensitive MAPK pathways^8^ and transcription factors such as AP-1, Nrf-2, and NF-kB, (g) the induction of DNA damage, (h) proinflammatory and profibrotic response in fibroblasts, and (i) the upregulated expression of metallothioneins and heme oxidase 1.

Some studies have described impaired in vivo wound healing when using silver-containing wound dressings and devices. Silver sulfadiazine (SSD) retarded the healing of burns in rats^9^, mice^10,11^ and rabbits^12^. A systematic review and meta-analysis of burns in children revealed that SSD therapy was less effective than non-silver alternatives^13^. SSD use was also connected to acute renal failure in burns patients^14^. Delayed epithelization in an ex vivo wound model and in vivo murine model was observed for several silver-containing wound dressings^15^.

Despite its popularity, SSD is regarded as obsolete and is being substituted with newer silver forms that are claimed to be less toxic. We, therefore, wanted to evaluate such claims by testing various forms of silver (including SSD) from selected wound healing dressings. Also, there is insufficient data on whether silver from dressings can penetrate skin tissue and concomitantly damage residing cells. We compared four commercially available silver-containing dressings used for the treatment of chronic wounds. The tested dressings contained silver in different forms: salts (Ag^+^), nanoparticles or nanocrystals (Ag^0^), or complex molecules such as SSD. Our study combined ex vivo (skin explants and full peripheral blood) and in vitro (cell cultures) investigations to assess the toxicity and efficacy of commercially available silver dressings.

## Results

### Characterisation of the tested dressings

The tested dressings differed in both their construction and the chemical composition of their active layer. Aquacel Ag Hydrofiber (Aq), Acticoat (Ac), and Silvercel Hydroalginate (Sc) are partially or entirely composed of non-woven textile which acts as an absorbent. Ialugen Plus (Ia) is, on the other hand, a cotton mesh impregnated with silver sulphadiazine cream (Table 1).

**Table 1:** The ICP quantification of silver content in dressings and their extracts extracted into physiological saline or FBS-supplemented culture medium.

Dressing	Silver content in dressings (μg/cm^2^)	Silver content in saline extracts (μg/mL)	Silver content in medium extracts (μg/mL)
Aq	120^a^	0.41 ± 0.32	18.1 ± 5.6
Ac	919.1 ± 141.4	0.28 ± 0.10	21.8 ± 4.4
Sc	863.8 ± 26.8	0.43 ± 0.21	3.5 ± 0.4
Ia	120^a^	0.45 ± 0.19	45.9 ± 23.0

^a^Stated by the manufacturers.

The dressings also differ in their silver forms. The ionic silver in Aq was dispersed as separate crystals while the ionic silver in Sc created a diffused coating (Supplementary Fig. 1A,B). Ac contained densely-packed nanocrystalline silver nanoparticles (10 nm on average), (Supplementary Fig. 1A). The SSD cream in Ia was spread uniformly over the cotton mesh.

**Figure 1:**
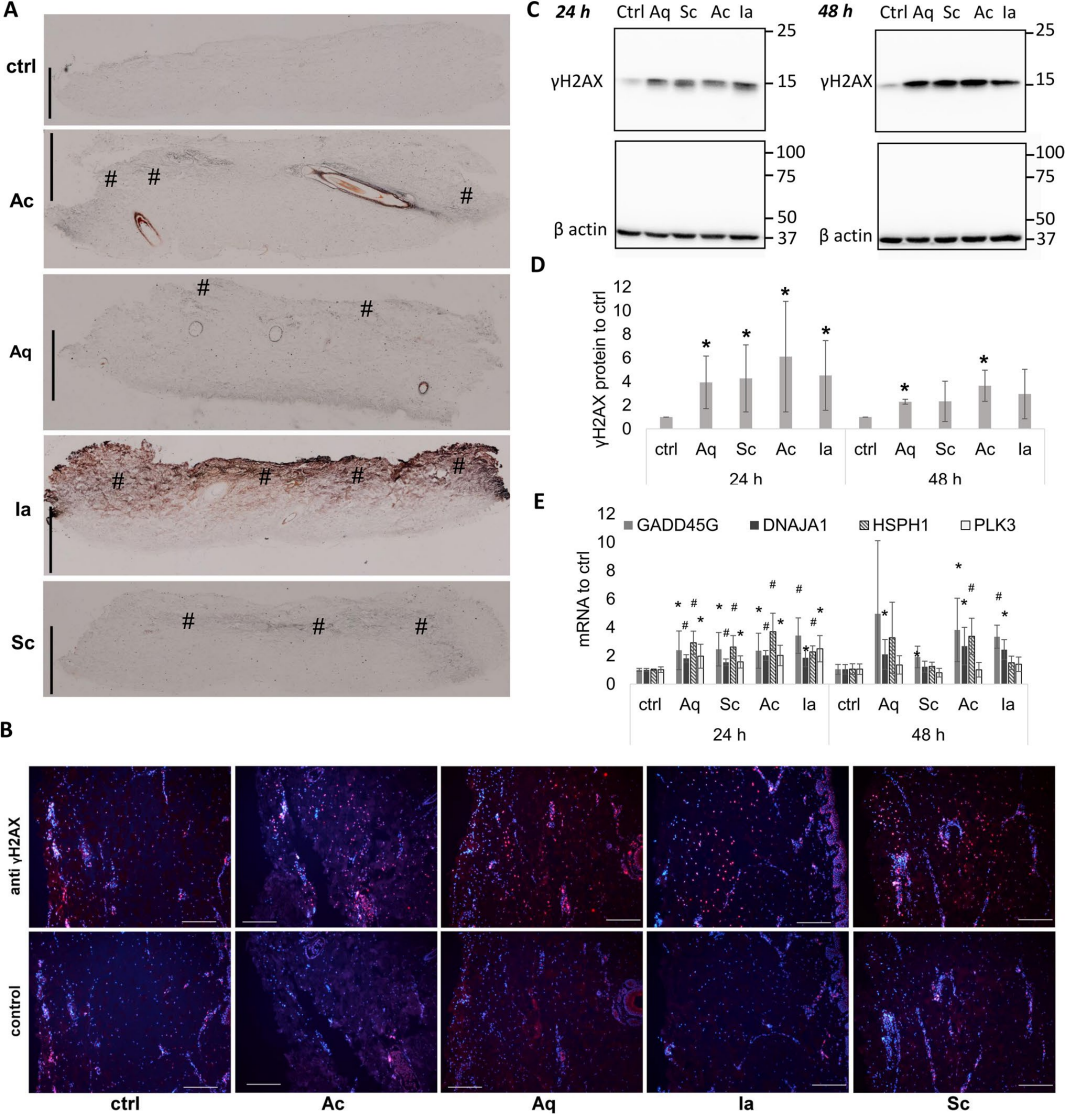
DNA damage in skin explants resulting from silver-containing dressings. Skin explants were treated with silver dressings or gauze (control) for 24 or 48 h. (**A**) Cross section of skin samples showing silver depositions highlighted using silver autometallography. Dressings were placed on the dermal sides of explants (this corresponds to the top of each sample image) and incubated for 24 h. Brown/black colour indicates silver depositions in skin tissue. The increased silver staining is marked with (#). Bars represent 1 mm. (**B**) The first row shows γH2AX (red) staining in explants treated with the dressings for 24 h; the second row shows controls without the primary antibody. Nuclei were counterstained with DAPI (blue). Bars represent 200 μm. (**C**) A representative Western blot image of γH2AX and the housekeeping protein β actin from skin explants incubated with silver-containing dressings for 24 or 48 h. The image is a composition of four Western blot membranes as indicated by dividing lines. (**D**) Quantification of γH2AX protein levels from 6 independent experiments treated as in (**A**). Mean ± SD. *p < 0.05, Student t-test relative to control in the specified time. (**E**) Relative expressions of GADD45G, DNAJA1, HSPH1, and PLK3 genes in silver dressing-treated skin explants from six independent experiments after 24 and 48 h. Mean ± SD. *p < 0.05, ^#^p < 0.001, Student t-test relative to control in specified times.

Not all the manufacturers clearly stated the amount of silver in their dressing. This information was available only for Aq and Ia (both contain 0.12 mg of silver ions per 1 cm^2^ of dressing), and, thus, we measured the silver content in Ac and Sc using ICP-OES (Supplementary Table 1). Also, we quantified the amount of silver that was extracted into physiological saline or culture medium with FBS. The dressings differed widely in their silver content, and the amount of silver in the extracts did not depend on the content of silver in the dressings (Table 1). While the amount of silver released into physiological saline was minimal and almost uniform among the dressings, more silver eluted into a cell culture medium supplemented with fetal bovine serum.

As one of the reported mechanisms of action of silver-based antimicrobial products is the generation of ROS, we tested the ability of the selected dressings to produce ROS directly (in cell-free conditions) by means of the HRP-catalyzed transformation of TMB to a coloured product. TMB is oxidised to the coloured product by hydroxyl radicals generated by HRP from H_2_O_2_^16^. Thus, this reaction can detect either hydroxyl radicals or hydrogen peroxide generated in the presence of the dressing in the solution. In this setting, only Ac generated detectable levels of ROS (Supplementary Fig. 2). To further investigate how fast the capacity of Ac to generate ROS diminished, the same sample of Ac dressing was transferred to a fresh reaction solution every 15 min. The amount of ROS generated during this experiment decreased gradually with every transfer and after the seventh exchange (105 min), almost no activity remained (Supplementary Fig. 2B).

**Figure 2:**
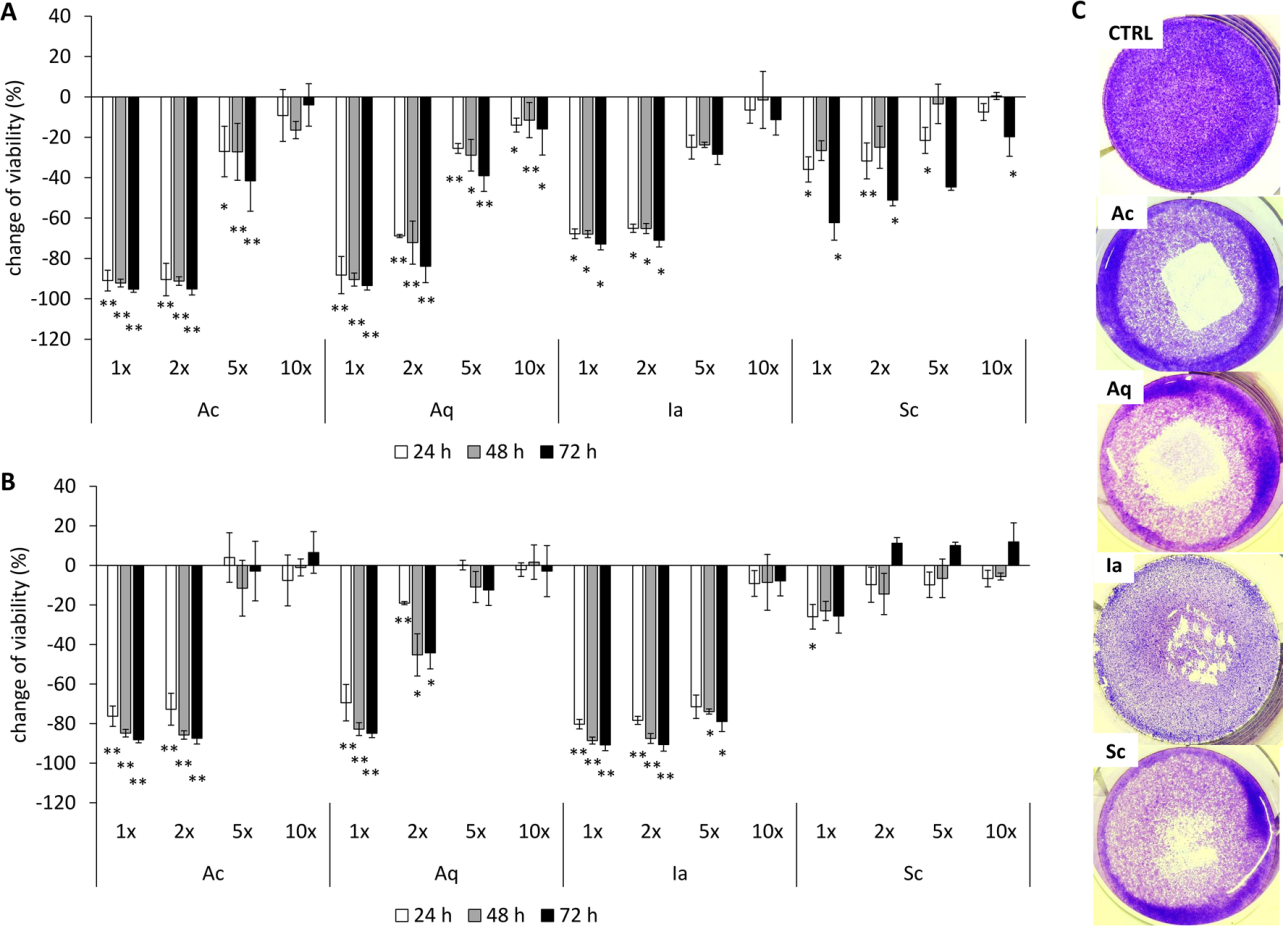
Cytotoxicity and contact inhibition of silver dressings. Change in viability of HaCaT keratinocytes (**A**) or NHDF (**B**) after 24, 48, and 72 h of treatment with dressing extracts diluted one to tenfold in a cultivation media measured by MTT assay. Bars represent mean of three independent experiments ± SD. **p < 0.01, *p < 0.05. (**C**) Contact inhibition of HaCaT cells after incubation for 6 h with pieces of dressings. White zones mark areas of dead cells. Images are representative of three independent experiments.

### Effects of silver penetration in skin explants

The ability of silver to penetrate into and through skin and its ability to cause genotoxic stress was evaluated in porcine auricle skin explants. The epidermis was removed by the heating method. When the silver dressings covered the skin explants in Franz diffusion cells, silver both penetrated inside and through the skin into the acceptor solution (Table 2). The average amount of silver accumulated in the skin after 24 h was similar for Aq, Ac, and Sc. Ia-treated skin samples had the highest silver content (though variable, likely due to inherently uneven SSD cream distributions), yet this content did not increase further during the next 24 h. After 48 h, Aq and Ac dressings released even more silver that penetrated into the skin. The least amount of accumulated silver in both skin and acceptor solution was detected for Sc, which is surprising when we consider the relatively high silver concentration in the Sc dressing itself. We further evaluated whether the silver penetration into the dermis was connected with DNA damage and stress response. Viable skin explants with the epidermis (to avoid tissue stress by epidermis removal) were cultivated with the dermal side up and with a piece of the tested dressing covering the skin surface for 24 or 48 h. Afterwards, part of each skin sample was processed for histological evaluation. As can be seen from silver autometallography staining, silver was detected predominantly on the dermal parts of explants which had been in contact with dressings (the upper parts of the histological sections); however, the intensity of silver staining differed significantly (Fig. 1A). The highest level of silver staining was observed for Ia, followed by Ac; Aq and Sc, which exhibited approximately the same levels; and control. This order of silver staining intensity roughly corresponds to the order of the amount of silver that penetrated from dressings into skin in Franz diffusion cells. Nevertheless, the autometallography method may enhance the silver signal from a silver salt (in Ia) seemingly more than from silver nanoparticles, as the same amount of silver may be dispersed more evenly when in the form of a salt.

**Table 2:** Silver penetration through de-epithelised skin explants.

Dressing	Silver content in skin [μg/g]		Silver content in acceptor fluid [μg/mL]	
	24 h	48 h	24 h	48 h
Aq	106.8 ± 49.9	125.3 ± 87.4	6.6 ± 8.7	24.3 ± 17.9
Ac	143.5 ± 43.4	293.7 ± 110.3	5.1 ± 3.2	3.6 ± 1.0
Sc	111.7 ± 62.3	86.6 ± 65.9	2.0^a^	3.5 ± 3.9
Ia	188.3 ± 161.8	197.6 ± 165.1	11.8 ± 13.2	11.0 ± 9.7

The skin was treated with silver-containing dressings in Franz diffusion cells for 24 or 48 h. Silver concentration in the skin or acceptor fluid was measured by means of ICP-OES. Mean ± SD, n = 3.

^a^Samples were below the limit of detection in 2 of 3 measurements.

Using immunofluorescence, we also stained the sections for γH2AX, a DNA double-strand break marker (Fig. 1B). As shown in the images, the nuclear signal of γH2AX increased in the explants treated with silver dressings compared to the control sample. Also, a DNA damage signal was apparent in cells “shielded” from direct contact with the dressings by the extracellular matrix. To quantify the γH2AX signal, we processed another part of each dressing-treated sample for Western blot (Fig. 1C,D); the results corresponded to the immunohistological observation and showed that DNA damage was significantly increased (p < 0.05) for all tested silver-impregnated dressings after 1 day and for dressings Ac and Ag after 2 days.

To broaden the scope of our analysis, we assessed the expression of selected genes in dressing-treated skin samples (Fig. 1E). The genes are known to be upregulated under cellular stress induced by various stimuli (PLK3, GADD45G) or play a role in heat shock response (DNAJA1, HSPH1). After 24 h of incubation, a significant increase (p < 0.05) in the expression of all selected genes was detected for all skin samples incubated with silver-impregnated dressings. After 48 h, the expression of at least one of the selected genes was significantly increased (p < 0.05) in each silver dressing-treated sample relative to control.

### In vitro cytotoxicity

We further evaluated different aspects of the toxicity of silver from the wound dressings using primary human dermal fibroblasts (NHDF) and the human HaCaT keratinocyte line. First, we determined the cytotoxicity of silver that eluted into an FBS-supplemented culture media using an MTT assay (Fig. 2). All tested dressing extracts were significantly (p < 0.05) toxic to HaCaT keratinocytes, with the order of toxicity as follows: Aq ≈ Ac > Ia > Sc. In fibroblasts, the toxic effect was significant for Aq, Ac and Ia dressing extracts while Sc was only slightly toxic after 24 h; the order of toxicity was as follows: Ia > Ac > Aq > Sc. The toxic effect was also proven for HaCaT cells that were in direct contact with dressings (Fig. 2C), the white zones marking dead, washed-out cells that were under the dressings or in the proximity zone. The overall lower staining intensity (most pronounced in Ia-treated cells) is a sign of a general decrease in viability. The same assay was performed using NHDF cells; however, due to the weak staining of these cells, the data are not presented here.

### DNA damage and oxidative stress in vitro

**Figure 3:**
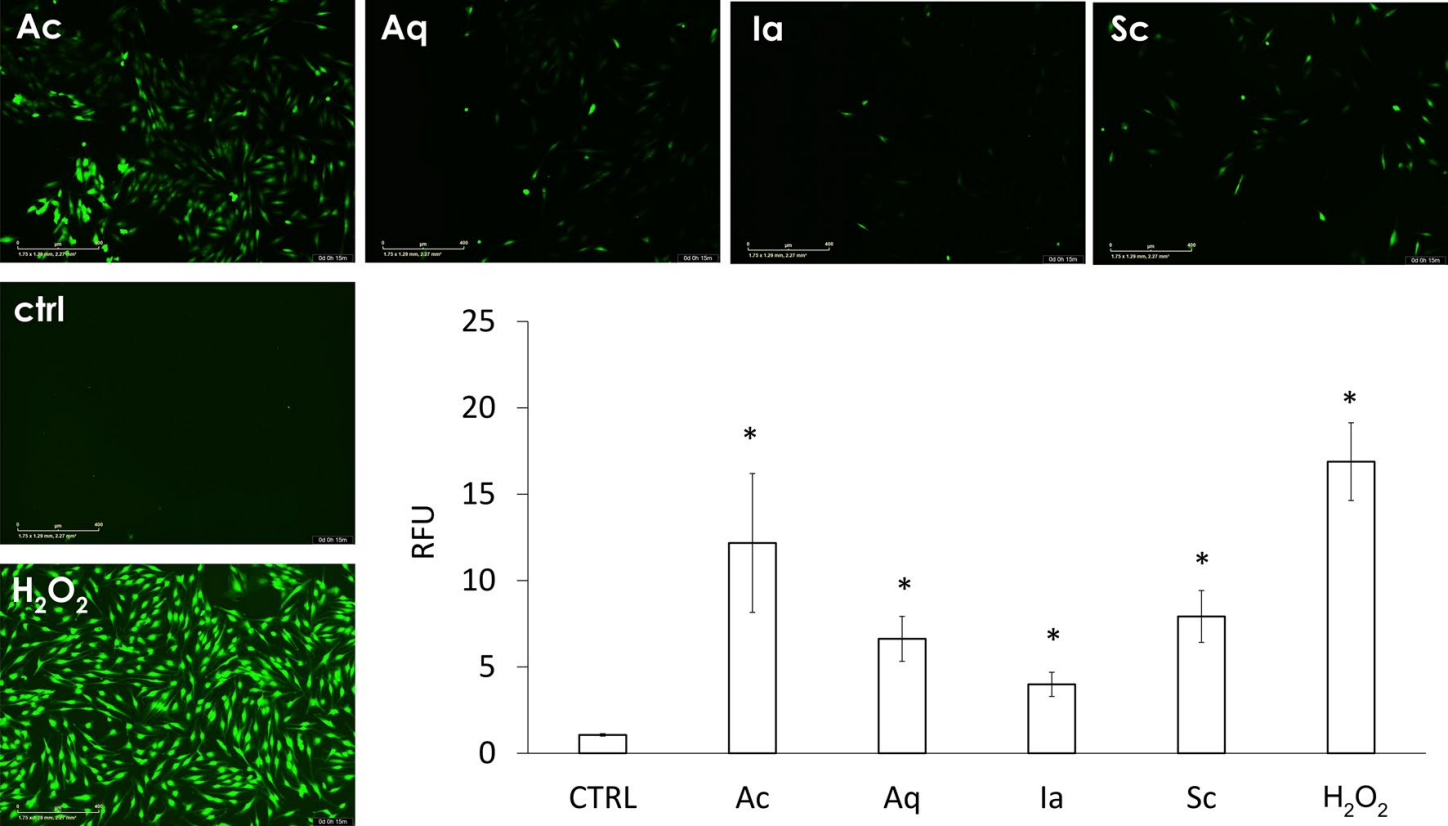
Intracellular oxidative stress. DCF-DA pre-labeled NHDF were incubated for 15 min with dressing extracts, or with 5 mM H_2_O_2_ as a positive control. Scale bar = 400 μm. Images are representative of 3 independent experiments. The graph shows the quantification of intracellular DCF fluorescence. Results are representative of means from three independent experiments ± SD. *p < 0.05.

Oxidative stress and subsequent DNA damage could have caused the observed toxicity. To evaluate oxidative stress, fibroblasts that were preincubated with the DCF-DA intracellular oxidative stress probe were treated with dressing extracts (elution for 72 h). DCF staining after 15 min showed that increased oxidative stress was mainly in fibroblasts treated with Ac, while Aq, Sc, and Ia elevated oxidative stress only slightly (Fig. 3). This observation corresponds with the measured immediate direct ROS-generating capacity of the silver dressings.

**Figure 4:**
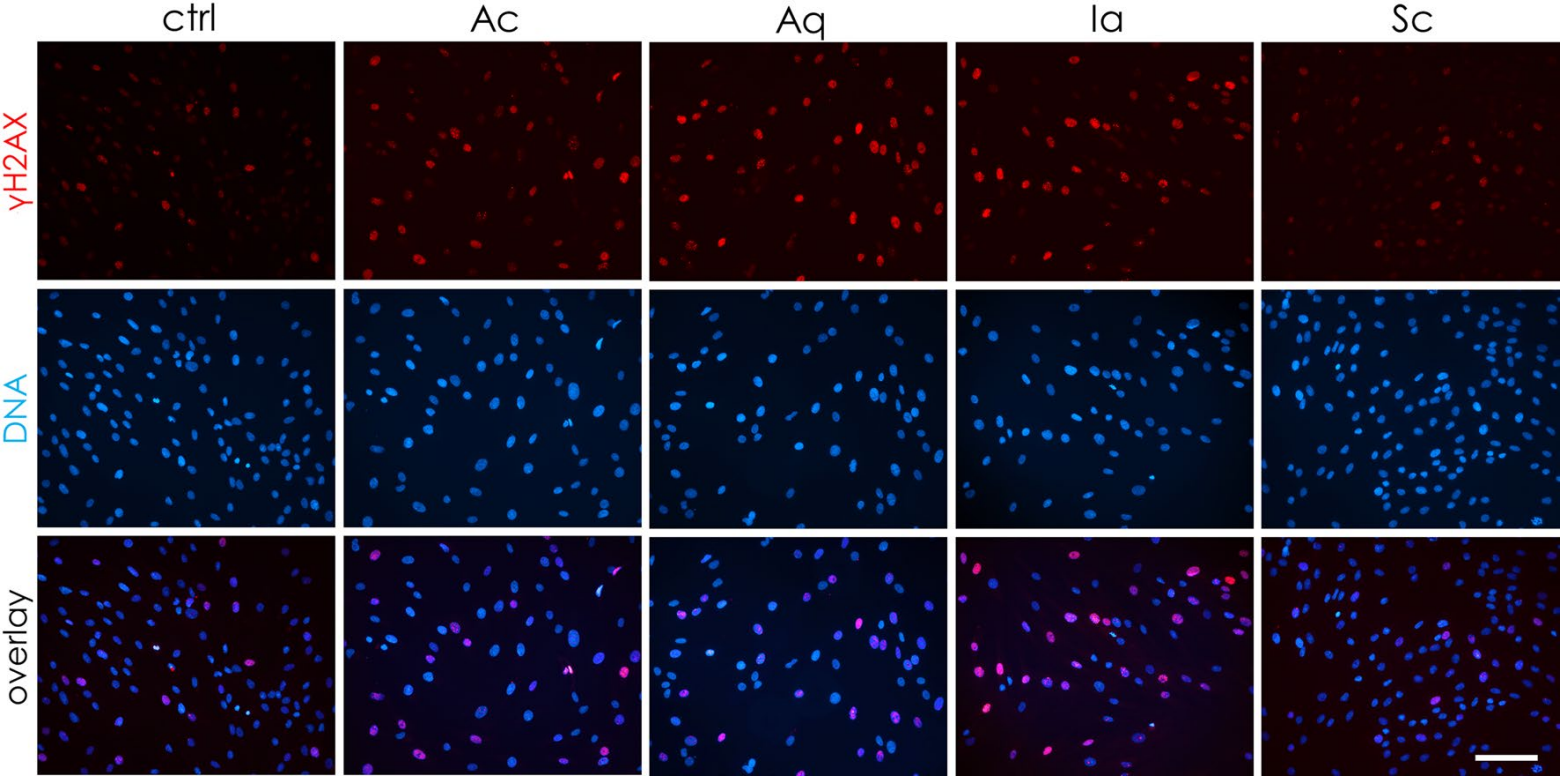
Immunocytochemical detection of DNA damage in NHDF cells after treatment with silver-containing dressing extracts. DNA damage marker γH2AX (red), DNA stained with DAPI (blue), and channel overlay. Scale bar = 100 μm. Images are representative of three independent experiments.

DNA damage was evaluated after 24 h incubation with dressing extracts. Then, the cells were stained for the DNA damage marker γH2AX. In Fig. 4, an increase in γH2AX staining is apparent mainly in Ac-, Aq- and Ia-treated fibroblasts, while Sc exhibited similar levels of γH2AX staining as the untreated control. As can be seen, nuclei of Ac-, Ag- and Ia-treated cells are enlarged in comparison to control and Sc-treated cells.

### Effects on immune cells

**Figure 5:**
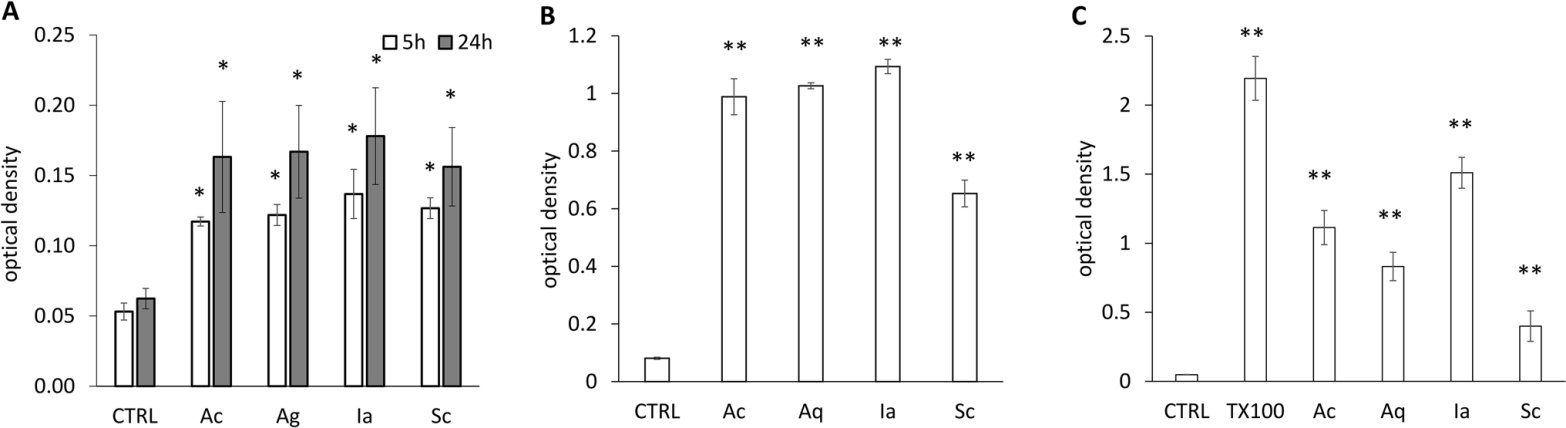
Effect of silver dressings on neutrophil activation. (**A**) Non-activated or (**B**) PMA-, and (**C**) OZP- pre-activated neutrophils were incubated with dressing extracts (in 10% FBS RPMI-media) and their oxidative burst measured. The results represent means of four independent experiments ± SD, *p < 0.05, **p < 0.01.

Silver dressings are reported to inhibit leukocyte (neutrophil) activation and reactivity. This feature is considered beneficial as it may dampen the excessively increased inflammation in chronic wounds. Neutrophil activation, also understood as ROS production or the oxidative burst of neutrophils, was measured in human peripheral whole blood. All tested dressing extracts (in FBS-supplemented RPMI medium) inhibited pre-activated neutrophil oxidative burst, while they had no significant effect on the spontaneous oxidative burst of non-activated cells (Fig. 5). As the inhibition of already stressed pre-activated neutrophils can be caused by non-specific toxicity in whole blood, lactate dehydrogenase (LDH) release from damaged cells was detected in isolated neutrophils treated with the same extracts as in the whole blood testing. Supplementary Fig. 3A shows that LDH levels were significantly increased in all tested samples.

**Figure 6:**
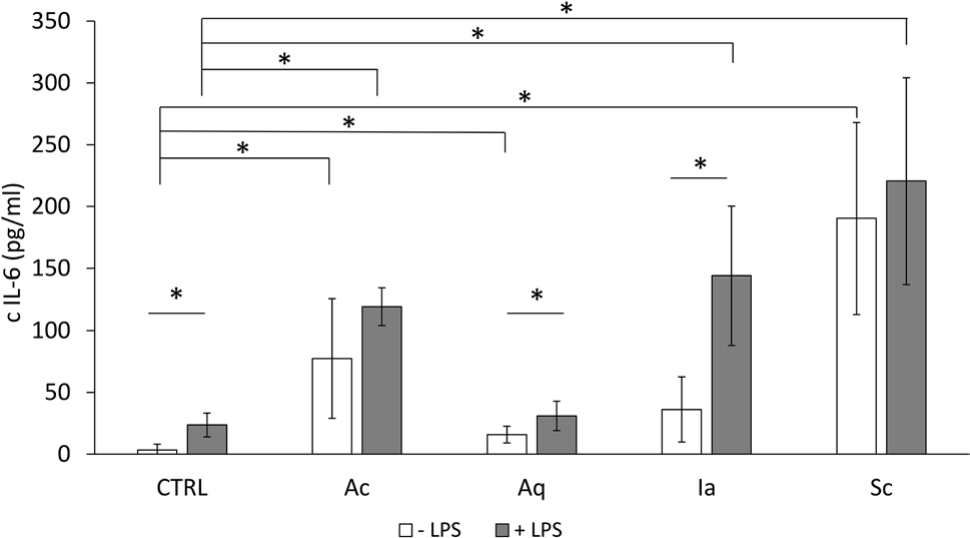
Monocyte activation test with pieces of silver-impregnated dressings. Production of IL-6 cytokine by whole blood monocytes after 16 h incubation with the dressings. White columns represent samples without LPS, grey columns show samples with added LPS (0.25 IU/mL). Error bars show means of three independent experiments ± SD, *p < 0.05.

Nevertheless, neutrophils are a minor leukocyte type present in chronic wounds^17,18^; monocytes/macrophages are the most abundant type of chronic wound immune cells. As monocytes are known for their ability to respond to foreign material, we tested whole dressings instead of dressing extracts in a monocyte activation test (MAT). In Fig. 6, the levels of pro-inflammatory IL-6 are reported for non-activated monocytes and LPS-activated cells incubated for 16 h with the dressings. Interestingly, all dressing-treated samples increased IL-6 release, which was statistically significant (p < 0.05) for Ac, Aq and Sc. In LPS-activated monocytes in blood, Ac, Ia and Sc significantly (p < 0.05) increased IL-6 release when compared to LPS alone. When comparing non-activated and LPS-activated blood, an increase was apparent for all dressings, although significant elevation was detected only in Aq- and Ia- treated blood.

Hemolysis was observed during the MAT. We, therefore, quantified the hemolysis post MAT using Triton X-100 as a positive control. A significant increase in absorbance (the content of free haemoglobin in a sample) was detected for all dressings (Supplementary Fig. 3C). Approximately half of all erythrocytes lysed during 16 h of incubation with the dressings. The most pronounced hemolysis was observed in the Ia-treated sample, while Sc induced the least hemolysis. Moreover, increased LDH release was also detected in the MAT samples (Supplementary Fig. 3B), suggesting a damaging and toxic effect of the dressings on blood cells. Similarly to hemolysis, all dressings caused significant (p < 0.05) LDH release; Sc was again the least damaging.

### Antibacterial activity

The antibacterial efficacy of the dressings was evaluated first by the standard agar diffusion method using the common wound pathogens *Pseudomonas aeruginosa* and *Staphylococcus aureus*. Zones of bacterial growth inhibition were present around all tested dressings (Supplementary Fig. 4). Ia was the most effective in inducing the largest inhibition zone. No major differences in the antibacterial effectiveness of the dressings were observed between Gram-positive *S. aureus* and Gram-negative *P. aeruginosa* in this test.

The dressings were extracted to an FBS-supplemented medium to better model silver elution into wound fluid. The dilution of extract that prevented measurable bacterial growth was considered as the minimal inhibitory concentration (MIC). Supplementary Table 2 shows that the antimicrobial effects of the dressing extracts corresponded to a certain degree to the silver concentration in the extracts, except for Aq; the Sc extract was as antimicrobial as Aq, which contained five-fold more silver. In contrast, Sc was less cytotoxic towards skin cells than Aq. Ia and Ac extracts inhibited the growth of *S. aureus* and were twice as effective as Sc or Aq against *P. aeruginosa*. Ia, which contained twice as much silver as Ac, performed similarly to Ac.

## Discussion

All tested dressings are used in the treatment of infected wounds and contain different forms of silver as an antimicrobial component. The manufacturer stated the amount of silver only for Aq and Ia (Supplementary Table 1). For Ac, our data were in good agreement with the previously reported values of 0.8–1.3 mg/cm^2^^19^. The dressings differed not only in their silver content but also in their construction (Supplementary Fig. 1, Supplementary Table 1), which can influence silver release and thus their effectivity and toxicity. Moreover, the composition of the extraction medium is also critical, as the silver contents in normal saline and FBS-supplemented media extracts of dressings were vastly different. Silver release into saline was limited and comparable among the dressings. However, extraction was more effective and diverse in protein-rich medium (Table 1). Silver-protein complexes may form, which increases silver solubility in body fluids^20^. Surprisingly, the measured amount of released silver did not correlate with the silver content in the dressings.

The ability of silver to penetrate through the dermal extracellular matrix was evaluated using porcine skin, which has a similar structure to the human dermis. However, the composition of wound granulation tissue is different from intact dermis. The amount of silver detected in the skin, or the acceptor solution (Table 2), did not directly correlate with the silver concentration in dressings and corresponded more with the silver content extracted into the protein-rich medium. Also, the amount of silver detected in the FBS-supplemented medium correlated with the cytotoxicity and γH2AX staining. SSD is considered to penetrate poorly through the skin, and penetration enhancers are used^21^. The high content of glycerol in Ia could have increased SSD penetration in our experiments. Several studies have already shown that silver (as Ag^0^ or Ag^+^) can penetrate through damaged skin and reach circulation in vivo^22–24^ or ex vivo^25^. In addition, Larese et al. observed the detectable penetration of silver from AgNP applied on intact ex vivo human skin^26^.

Silver deposits in skin explants were detected using autometallography in histological sections. Silver accumulated primarily on the tissue border after Sc treatment, but was visible in deeper layers, often surrounding blood vessels, after treatment with the other dressings (Fig. 1A). Fredriksson et al. observed a similar pattern of staining in an ex vivo human wound model treated with Aq, Ac, SSD, or AgNO_3_^27^. Silver penetration correlated with the increased staining of γH2AX throughout the whole dermis (Fig. 1A). The induction of this marker of oxidative stress and DNA damage by silver was described in vivo^28,29^ and in vitro^30^. Zhao et al. observed in vitro that the amount of γH2AX was directly proportional to the silver dose^31^*.* In our ex vivo experiment, the silver dressings caused an increase in γH2AX (Fig. 1B,C), which corresponded with similar levels of silver accumulated inside the skin explants. The stress response of tissue was also accompanied by an increase in damage-responding and chaperon-coding genes (Fig. 1D). These genes have already been linked to silver-induced DNA stress. An increase in the expression of GADD45G^32^, DNAJA1^33^ and HSPH1^34^ after AgNP treatment was observed in cancer cells. PLK3 is known as a cell cycle regulator, and its negative role in corneal healing was reported by Lu et al.^35^. We observed the silver from wound healing dressings to have similar effects in skin explants.

Ex vivo toxicity data was compared to the toxicity assessed in vitro using relevant wound cell types—namely, HaCaT keratinocytes and primary human dermal fibroblasts. By using the MTT method, we showed that undiluted extracts induced a highly acute toxic response in both keratinocytes and fibroblasts (Fig. 2). Eluate from the Sc silver dressing was the least toxic, which corresponds with the Sc extract exhibiting the lowest silver concentration. Probably due to their higher proliferation rate, keratinocytes were more sensitive to extracts except in the case of the lower dilution of the Ia extract, which was more toxic to fibroblasts. Similar results were obtained during AgNP testing on primary fibroblasts and keratinocytes, where keratinocytes also exhibited higher sensitivity^36^. The toxicities of the dressing extracts correlated with direct contact inhibition in the cell monolayer. The pattern of cell staining showed that Ac-treated cells were dead directly under the dressing, while Aq treated cells showed a halo zone of dead cells around the dressing (Fig. 2C). This difference might be the result of a different rate of silver release from Ag^0^ and Ag^+^ containing dressings.

As silver toxicity is most often connected to DNA damage via increased oxidative stress, we evaluated both these parameters in dermal fibroblasts treated with dressing extracts. In good agreement with our toxicity assessment, we observed that only the Sc dressing failed to induce significant DNA damage in vitro (Fig. 3). The enlarged nuclei observed in all dressings except for Sc also suggest that subtoxic concentrations of dressing extracts may induce senescence^37^, which can be potentially even more harmful to wound regeneration due to the pro-inflammatory phenotype of senescent cells^38^. The DNA damage is probably linked to the increased oxidative stress, which we visualised using DCF-DA (Fig. 4). Similarly to Harvanova et al.^39^, we observed a rapid increase in intracellular ROS level for Ac containing Ag^0^; the rest of the tested dressings (containing Ag^+^ or SSD) showed only a mild increase. Also, cell-free detection showed primary ROS production only for Ac (Supplementary Fig. 2).

Silver dressings are reported to decrease immune cell activity and are thus considered anti-inflammatory^40–42^. Only a few reports of anti-inflammatory effects concern toxicity and cellular damage to inflammatory cells^43^. We observed both immune cell inhibition and activation. All tested dressings were able to significantly decrease oxidative burst in the activated neutrophils (Fig. 5). Surprisingly, no effect of silver dressings was observed on non-activated neutrophils, suggesting that the silver inhibitory effect is dependent on the already activated pro-inflammatory state. Such anti-inflammatory effects can be at least partially explained by the toxicity of silver, as we detected a significant release of LDH from neutrophils concomitantly with a decrease in pro-inflammatory oxidative burst (Supplementary Fig. 3A). In addition, Tautenhahn et al. reported that silver-coated polyester grafts disrupted neutrophil functions and tissue regeneration^44^. Therefore, further studies should investigate how silver dressings affect immune cells in respect of their microbicidal and regenerative activity. In contrast to the anti-inflammatory effect in neutrophils, full blood incubated with the dressings showed an increase in the production of proinflammatory cytokine IL-6, and a similar effect was present also in LPS-activated monocytes (Fig. 6). These results corroborate previous observations^45^. As in neutrophils, we detected cytotoxicity expressed as LDH release in full blood, which could be connected to haemolysis^46^.

To correlate the cytotoxic effects of the tested dressings with their efficacy, we measured antimicrobial activity using the microdilution method. As the MIC for the dressings containing an ionic form of silver (Sc and Aq) differed markedly, other factors may influence antimicrobial efficacy. Using the microdilution method, gram-positive *S. aureus* was less sensitive to silver extracts than gram-negative *P. aeruginosa*, which is consistent with previous results^4, 47^.

In summary, the toxicity of the silver dressings was only partially driven by the silver content of the dressings and more so by the ability to release Ag^+^; Burd et al. came to a similar conclusion^15^. Interestingly, the least toxic dressing (Sc) performed well in the antimicrobial assay, suggesting that antimicrobial effectivity and cytotoxicity are not directly linked. While the low toxicity of Sc could be an effect of the alginate, which has antioxidant properties^48^, the same effect was not observed in other dressings (Aq and Ia) containing the polysaccharides carboxymethylcellulose and hyaluronic acid, respectively. Future research could explore the reciprocal effects of silver (or alternative antiseptics) combined with pro-healing (bio)molecules on wound healing. It is also important to measure other parameters of antiseptics than cytotoxicity, as subtoxic levels of silver were still able to induce intracellular ROS production and DNA breaks. These effects can be detrimental particularly in rapidly proliferating cells during the granulation and reepithelization phases of wound healing. Moreover, we did not observe the claimed anti-inflammatory effect of silver in whole blood. The oxidative burst of pre-activated neutrophils was most likely inhibited by silver toxicity. In addition, the silver dressings produced a simultaneous increase in the levels of IL-6 and haemolysis in whole blood. We showed that silver could penetrate skin ECM and damage the residing cells. We investigated the effects in skin only after 1 or 2 days, which is a relatively short time period compared to the weeks and months often necessary for the treatment of non-healing wounds, during which silver is periodically supplied to the wound.

## Conclusion

In this study, we evaluated four dressings used in the treatment of chronic non-healing or hard-to-heal wounds. We investigated the physicochemical properties of silver contained in wound dressings and the induction of selected toxic and antimicrobial processes in vitro and ex vivo by these dressings. To our knowledge, this is the first time that such a broad selection of characterisation methods was employed to compare the toxic effects of commercially available wound healing products. That is, we broadened observations of silver cytotoxicity from in vitro settings to ex vivo*.* Also, to our knowledge, we show for the first time that silver released from dressings has the potential to diffuse into intact porcine dermis and induce DNA damage and stress response in the residing cells. While wound healing, and especially the healing of chronic wounds, is difficult to model accurately in vitro and even ex vivo, we believe that our approach provides significant and novel information about the mechanisms of silver activity, which can be extrapolated to wound healing. It is essential to use a complex methodology to test the efficiency as well as safety of wound dressings, as these properties are related but not directly proportional. Also, silver dressings simultaneously affect the viability of bacteria and wound inflammatory, stromal, and epidermal cells. Moreover, our data support recent recommendations that silver dressings should be applied to only highly infected non-healing wounds and not to well-managed and already healing wounds, where silver toxicity can be detrimental to proliferating cells.

## Methods

All methods were carried out in accordance with relevant guidelines and regulations. Informed consent was obtained from all subjects.

### Material

The following silver dressings were compared: Acticoat (Smith&Nephew, UK; referred to in this article as Ac), Aquacel Ag + Extra (Convatec, UK; referred to in this article as Aq), Silvercel Hydro-Alginate (Systagenix, UK; referred to in this article as Sc), and Ialugen Plus (IBI, Czech Republic; referred to in this article as Ia).

Other chemicals were obtained from Sigma–Aldrich (St. Louis, USA) if not stated otherwise.

### Preparation of extracts

In several assays, extracts from dressings were used rather than the dressings themselves. These extracts were prepared using physiological saline (0.9% (w/v) NaCl) or cell culture media supplemented with 10% fetal bovine serum (FBS). The ratio of the dressing area to the solution was 1 cm^2^ per 4 mL of solution. Extraction was performed for 72 h at RT with constant agitation. The extracts were sterilised by passing them through a 0.2 μm filter and stored at 4 °C until use.

### Silver concentration measurement

The silver concentrations in dressings and the extracts of dressings were measured using ICP-OES. The silver concentration was also evaluated in porcine ex vivo skin samples. 10–70 mg of each sample was transferred to a PTFE vessel for microwave digestion. Then, 1 mL of concentrated nitric acid (trace analysis grade, Analytika Ltd., Czech Republic), 0.8 mL of concentrated hydrochloric acid (trace analysis grade, Analytika Ltd., Czech Republic) and 0.2 mL of hydrogen peroxide (p.a. 30%, Penta Ltd., Czech Republic) were added. The sample was digested at 150 °C for 5 min and then, in the second step of the same cycle, at 200 °C for 10 min. After digestion, the sample was quantitatively transferred using 0.2 mL of hydrogen peroxide and deionised water.

The analysis was performed with an ICP-OES instrument (Radial 725, Agilent Technologies, Australia) equipped with a pneumatic Sea-spray nebuliser and cyclonic spray chamber. Quantification was based on external calibration. Method accuracy and reliability were tested utilising porcine skin samples spiked with known concentrations of Ag from a certified reference solution (Analytika Ltd., Czech Republic).

### Scanning electron microscopy

Scanning electron microscopy (SEM) analyses were performed using a Zeiss Ultra Plus instrument (Zeiss, Germany). Samples were coated with a thin layer of Au/Pd in a Quorum SC7620 sputter coater. Images were taken by two secondary InLens and SE2 electron detectors for higher or lower magnification, respectively. Scanning parameters were as follows: accelerating voltage, 3.5 kV; probe current, 36 pA; and pressure in the chamber, ~ 7 × 10^–5^ Pa.

EDX images were captured with the Zeiss Ultra Plus instrument. X-ray maps and spectra were taken by an X-Max^N^ 80 X-ray detector (Oxford Instruments, UK). The EDX parameters were as follows: accelerating voltage, 10 kV; probe current, 300 pA; working distance, 8.5 mm; and process time, 4 [AU].

### Direct oxidative activity of dressings

The generation of ROS in cell-free conditions was evaluated using 3,3′,5,5′-tetramethyl-benzidine (TMB) as a substrate for horseradish peroxidase (HRP). Circles of diameter 6 mm were cut out of the dressings and tested. Briefly, the working solution consisted of 0.1 mg/mL TMB (stock solution c = 1 mg/mL in DMSO) mixed 1:9 with 0.05 M citrate–phosphate buffer (pH = 5) and HRP (final c = 0.16 IU/mL). Each piece of dressing was immersed into 0.5 mL of the working solution and samples (25 μL) were collected at 0, 5, 7.5, and 10 min. Immediately after collection, the samples were mixed in the ratio of 1:1 with 0.16 M H_2_SO_4_ and absorbance was read at 450 nm (reference wavelength = 540 nm) using a NanoDrop OneC instrument (ThermoFisher Scientific, US). 30% H_2_O_2_ was used as a positive control. The background signal (blank solution) was subtracted.

### Silver penetration into de-epithelized skin

We used static diffusion Franz cells to investigate silver penetration into de-epithelized skin. The skin was obtained from a local slaughterhouse (Bocus, Letohrad, Czech Republic) and excised from the inner part of the porcine auricle. Hair was shaven, the skin was incubated in PBS (60 °C, 90 s), and the epidermis was peeled off. The average thickness of the de-epithelized skin was 2 mm. The skin pieces were placed into a chamber and covered with a tested silver dressing or a piece of gauze. Each donor chamber was filled with 0.3 mL of NHDF culture medium with 10% FBS. The acceptor chamber contained 0.7 mL of culture medium. The diffusion cells were incubated at 37 °C for 24 or 48 h. After incubation, the skin was rinsed with PBS, and the parts of the skin that separated the donor and acceptor chambers were analysed for silver content using ICP-OES, as described above. The amount of silver that penetrated through the de-epithelized skin was measured in the donor fluid. Three independent replicates were prepared at both times.

### Cultivation of ex vivo skin with dressings

Fresh (1–2 h) porcine auricles (Bocus) were scrupulously cleaned with soap and Betadine (a PVP iodine solution, Egis Pharma, Hungary) and rinsed with water. The skin pieces (1 × 1 cm) from auricles were excised and placed into antibiotic solution (penicillin–streptomycin solution, 10,000 U/mL penicillin, 10 mg/mL streptomycin) for 30 min at 37 °C under atmospheric O_2_ and CO_2_. Skin samples with intact epidermis were placed dermal side upwards in 6-well cultivation plates with 3 mL of NHDF medium supplemented with 10% FBS and then covered with 1 × 1 cm of selected silver-containing dressing or sterile control gauze soaked with 300 µl of cultivation medium. The samples were incubated for 24 or 48 h. Afterwards, the skin was rinsed with PBS and each of the skin explants was divided into thirds for histological examination (4% PFA fixation at 4 °C), protein analysis by means of Western blot (snap freezing with liquid nitrogen), and qPCR (overnight in RNAlater (Thermo Fisher Scientific), then at − 80 °C).

### Histological staining

The autometallographic staining of silver was adopted according to Danscher et al.^49^. Images were captured using an Eclipse 50i microscope with an attached DS-Fi1 camera (Nikon, Yokohama-Shi, Japan).

For the immunofluorescence of γH2AX, histological sections on Superfrost Plus glass slides (Thermo Fisher Scientific, Waltham, MA, USA) were deparaffinized and incubated overnight with primary antibody (1:1,000, ANTI-PHOS-HIST H2A.X S139, clone JBW301, Millipore, MA, USA). They were then incubated for 1 h with a secondary antibody (ab150114, Abcam, Cambridge, UK; dilution 1:10 000) and mounted on slides with ProLong Diamond Antifade Mountant with DAPI (ThermoFisher Scientific). Images were captured with a Nikon Eclipse Ti (Nikon, Yokohama-Shi, Japan) fluorescent microscope.

### Gene expression

Porcine skin after incubation with dressings was processed for RNA isolation, cDNA synthesis, and qPCR gene expression as described previously by Klein et al.^50^ TaqMan Real-Time PCR Assays (Thermo Fisher Scientific, Waltham, MA, USA) used for qPCR were: DNAJA1, Ss04326380_g1; PLK3, Ss03375596_u1; HSPH1, Ss03388958_m1; GADD45G, Ss04246840_g1. RPL13A, Ss03376908_u1. Threshold cycle values were normalised to the RPL13A housekeeping gene and related to gauze control samples for the particular time (24- or 48-h interval) using the 2^−ΔΔCt^ method^51^. Six independent samples for each treatment and time were measured and averaged. The significance of differences in gene expression relative to gauze control was evaluated using Student’s t-test for every silver dressing in the specified time.

### Western blotting

For subsequent Western blot analysis, tissue lysates were prepared from frozen porcine skin. Using a scalpel in a drop of lysis buffer (50 mM Tris pH 8; 150 mM NaCl; 1% Triton X-100; 0.5% sodium deoxycholate; 1% sodium dodecyl sulfate; 4% urea), the skin was cut into small pieces, which were subsequently homogenized in 350 µl of the lysis buffer using a stainless steel 5 mm bead and a tissue homogenizer (TissueLyser II, Qiagen, Hilden, Germany) for 10 min at 25 Hz. Protein concentration was measured by means of BCA protein assay kit (Thermo Fisher Scientific).

Proteins in lysates were separated using 4–15% gradient SDS-PAGE and transferred onto polyvinylidene difluoride membranes. To detect specific proteins, membranes were incubated overnight in a blocking buffer (20 mM Tris; 137 mM NaCl; 0,05% Tween 20; 5% dried low-fat milk powder) with the primary antibody phospho-histone H2A.X (20E3) (1:1,000; Cell Signaling, US). β-actin was used as a protein quantity loading control (1:2000; Santa Cruz, US). The membranes were developed with HRP-conjugated anti-rabbit or anti-mouse Ig using chemiluminescent detection to visualise signals (Clarity Western ECL substrate; Bio-Rad, US). The signals were scanned and evaluated with an Alliance 9.7 Chroma Chemiluminescence Imaging System (UVItec Limited, UK).

### Antibacterial activity

The agar diffusion method was used to compare the antimicrobial activities of the dressings. A sample piece (1 cm^2^) of each dressing was incubated on a freshly inoculated Petri dish with either *Staphylococcus aureus* or *Pseudomonas aeruginosa;* these strains were isolated from human chronic wounds, characterised and cultivated as described previously^52^. Additionally, the antimicrobial effectivity of dressing extracts (prepared in RPMI medium with 10% FBS, as described above) was compared by means of the microdilution method^53^. Optical density was measured by an Ensight Multimode plate reader (PerkinElmer, Waltham, MA, USA) at 600 nm for 24 h (shaking, 37 °C).

### Cell culture

Normal human dermal fibroblasts from adult skin (NHDF) were purchased from Lonza (Basel, Switzerland). NHDF were cultivated in Dulbecco’s modified Eagles low glucose medium (DMEM) supplemented with 10% FBS, glutamine (0.3 mg/mL), glucose (4 mg/mL), penicillin (100 units/mL), and streptomycin (0.1 mg/mL) in 75 cm^2^ culture flasks under 5% CO_2_ and at 37 °C until the fifth passage. HaCaT keratinocytes were purchased from Hölzel Diagnostika (Köln, Germany) and were cultivated in the same way but without the addition of glucose to the medium.

### Viability measurement

Five thousand cells per well were seeded in a 96-well plate with 200 μL of the culture medium with 10% FBS in an incubator (37 °C, 5% CO_2_) overnight. On the following day, the cells were treated with 200 μL of a dilution series of extracts from the dressings (100%, 50%, 20%, or 10% of the original extract diluted with culture medium supplemented with 10% FBS) for 24, 48, and 72 h. Control cells were treated with a standard 10% FBS culture medium. Viability was measured as described previously^54^ using the 3-(4, 5-dimethylthiazol-2-yl)-2,5-diphenyl-tetrazolium bromide (MTT) assay. MTT stock solution (20 μL; c = 5 mg/mL) was added to the cell culture medium in each well. The plates were incubated for 2.5 h at 37 °C. Then, the MTT solution was removed, 220 μL of lysis solution (1:1 propan-2-ol with DMSO, 10% (w/v) Triton X-100 and 0.37% (w/v) HCl) was added, and lysis was carried out for 30 min at room temperature on a rotary shaker (150 rpm). The absorbance was read at 570 and 690 nm with an EnSight Multimode Plate Reader (PerkinElmer, USA). Data were processed in Kaleido (PerkinElmer, USA). The final absorbance was calculated as A = A_570_ – A_690_. The change in viability of treated cells relative to control cells was calculated as change [%] = (A sample/A control – 1) × 100.

### Direct contact inhibition assay

Cells were seeded at a density of 300 000 cells per well in a 6-well plate and incubated in the culture medium supplemented with 10% FBS at 37 °C and under 5% CO_2_ until confluence was achieved. Dressings were cut into squares of 1 cm^2^, placed on the cell layer, and incubated with the standard culture medium for 6 h. Control cells were treated with the medium only. Afterwards, the cells were washed with PBS and fixed with 4% paraformaldehyde for 15 min. The cells were stained with 0.1% (w/v) crystal violet solution (dissolved in 10% ethanol) for an hour and then rinsed with water. The inhibition zones were photographed using a Canon EOS 80D EF-S 18–55 mm f digital camera (Canon).

### Fluorescent staining of cells

For DNA damage analysis, NHDF cells were seeded on microscopic slides into a 6-well plate at a density 2 × 10^5^ per well and allowed to adhere overnight. On the following day, the cells were treated with 5 × diluted dressing extracts and incubated for 24 h. Subsequently, the cells were fixed using 4% PFA for 15 min. After permeabilisation and blocking, the slides were incubated with an anti-γH2AX rabbit primary antibody (Cell Signaling, US; 1:500 dilution in a blocking buffer—20% FBS, 0.3 M glycine, 0.05% Tween 20 in PBS; overnight; 4 °C). Detection was performed using a secondary antibody labelled with Alexa Fluor 555 (Abcam, UK; 1:500 in a blocking buffer; 1 h, RT). The slides were mounted using ProLong Diamond Antifade Mountant with DAPI (ThermoFisher Scientific). After the slides were dried, images were taken using an Eclipse Ti fluorescent microscope (Nikon, Yokohama-Shi, Japan).

Intracellular oxidative stress was detected using a DCF-DA probe, which is sensitive to the overall ROS concentration inside cells. The cells were seeded overnight in a 24-well plate and then labelled with DCF-DA (1 μg/mL) for 15 min, after which they were treated with 5 × diluted dressing extracts and incubated at 37 °C and under 5% CO_2_. 5 mM H_2_O_2_ was used as a positive control. The green fluorescence of DCF was recorded every 15 min during a 90 min period of incubation using an Incucyte S3 automated microscope (Essen Bioscience, USA). The quantification of intracellular fluorescence was performed in Fiji software following the method described by Čepa^55^.

### Determination of oxidative burst by neutrophils in whole blood

An independent ethics committee approved the blood sample collection for neutrophil oxidative burst assay and MAT (Ethical Committee for Research at Masaryk University, Brno, Czechia, approval no. EKV-2018-083). All donors gave their written consent.

The oxidative burst (ROS production) of blood phagocytes was determined in diluted whole blood by luminol-enhanced chemiluminescence (CL) using an LM-01T microplate luminometer (Immunotech, Czech Republic). Briefly, the reaction mixture consisted of 6 μL of whole blood in 54 µl of RPMI-1640 growth medium mixed with 60 µl of dressing extract in physiological saline or an FBS-supplemented medium. This mixture was incubated at 37 °C for 20 min. Just before the start of the measurement, we added 1 mM luminol (a stock solution of 10 mM luminol in a 0.2 M borate buffer) (Molecular Probes, USA). We determined spontaneous ROS production, and ROS production induced by opsonised zymosan particles (OZP—0.1 mg/mL) (Sigma-Aldrich, USA) or phorbol 12-myristate 13-acetate (PMA—0.8 µM; Sigma-Aldrich, USA). Untreated samples without the tested extracts were evaluated as controls. The assays were run in duplicates. Chemiluminescence was recorded continuously for 90 min at 37 °C and was expressed as relative light units (RLU). We determined the total amount of ROS production from the integrated area under the chemiluminescence curve.

### Monocyte activation test (MAT)

MAT was performed in 10 × saline-diluted heparinised human whole blood. Blood samples were collected from five healthy donors, pooled, and diluted with saline. The diluted blood samples were incubated with 6 mm-diameter circles cut from dressings in sterile microtubes for 24 h at 37 °C. In parallel, lipopolysaccharide (LPS, final c = 0. 25 IU/mL; Endotoxin Standard E-Toxate; Sigma, US) was added to the second series of samples incubated with dressing samples to stimulate an innate immune response to bacteria. Blood cells sedimented during the incubation, leaving an upper phase of saline-diluted plasma. After incubation, 200 μL of each saline-diluted plasma sample was collected and immediately assayed in duplicates for IL-6 using an IL-6 Human Uncoated ELISA Kit according to the manufacturer’s protocol (ThermoFisher Scientific, US). Absorbance was read using an EnSight Multimode Plate Reader (PerkinElmer, US) and the final IL-6 concentration was calculated by Kaleido SW (Perkin Elmer, US). The remaining plasma and sedimented cells were stored at 4 °C for the assessment of hemolysis and toxicity.

### Hemolysis

Hemolysis was detected in the remaining saline-diluted plasma and sedimented cells from MAT. After centrifugation, 100 μL of each sample was transferred to a 96-well microplate and absorbance was measured at 550 nm. 1% Triton X-100 was used as a positive control.

### LDH release

The toxicities of silver dressings to blood cells were evaluated by means of lactate dehydrogenase (LDH) assay (Cytotoxicity Detection KitPLUS, Roche, Switzerland) according to the manufacturer’s instructions. Two sources of blood cells were used—human whole blood incubated with silver dressings as in MAT, and isolated neutrophils. The absorbance used for background correction was measured in blank samples without the LDH reagent. Results are reported as absolute values of optical density compared to an untreated sample.

### Statistical analysis

If not stated otherwise, the Student t-test was used to evaluate the statistical significance of differences between samples and untreated controls.

## Supplementary information

Supplementary information.

## Data Availability

The data generated in the current study are available from the corresponding author on request.
