# Author Correction: Effects of wound dressings containing silver on skin and immune cells

**DOI:** 10.1038/s41598-021-83765-1

**Published:** 2021-02-17

**Authors:** Kristina Nešporová, Vojtěch Pavlík, Barbora Šafránková, Hana Vágnerová, Pavel Odráška, Ondřej Žídek, Natálie Císařová, Svitlana Skoroplyas, Lukáš Kubala, Vladimír Velebný

**Affiliations:** 1 Contipro a.s., Dolni Dobrouc 401, 56102 Dolni Dobrouc, Czech Republic; 2 Third Faculty of Medicine, Charles University, Prague, Czech Republic; 3 Faculty of Natural Sciences, Charles University, Prague, Czech Republic; 4 Department of Biophysics of Immune System, Institute of Biophysics of the Czech Academy of Sciences, Brno, Czech Republic; 5 Department of Experimental Biology, Faculty of Science, Masaryk University, Brno, Czech Republic; 6 International Clinical Research Center, St. Anne’s University Hospital, Brno, Czech Republic

Correction to: *Scientific Reports* 10.1038/s41598-020-72249-3, published online 16 September 2020

This Article contains errors, where the Tables and Figures are incorrectly referenced.

In the Results section, ‘Characterisation of the tested dressings’,

“Ialugen Plus (Ia) is, on the other hand, a cotton mesh impregnated with silver sulphadiazine cream (Table 1).”

should read:

“Ialugen Plus (Ia) is, on the other hand, a cotton mesh impregnated with silver sulphadiazine cream (Supplementary Table 1).

In the Discussion section,

“In good agreement with our toxicity assessment, we observed that only the Sc dressing failed to induce significant DNA damage in vitro (Fig. 3).”

should read:

“In good agreement with our toxicity assessment, we observed that only the Sc dressing failed to induce significant DNA damage in vitro (Fig. 4).”

In the same section,

“The DNA damage is probably linked to the increased oxidative stress, which we visualised using DCF-DA (Fig. 4).”

should read:

“The DNA damage is probably linked to the increased oxidative stress, which we visualised using DCF-DA (Fig. 3).”

Lastly, Figure 5 is a duplication of Supplementary Figure 3. The correct Figure 5 appears below as Figure 1.

**Figure 1:**
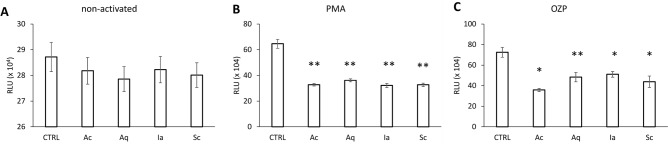
A corrected version of Figure 5. Effect of silver dressings on neutrophil activation. (**A**) Non-activated or (**B**) PMA-, and (**C**) OZP- pre-activated neutrophils were incubated with dressing extracts (in 10% FBS RPMI-media) and their oxidative burst measured. The results represent means of four independent experiments ± SD, *p < 0.05, **p < 0.01.

