# Supplementary material for: Effects of wound dressings containing silver on skin and immune cells
Source: Sci Rep. 2020 Sep 16;10:15216. doi: 10.1038/s41598-020-72249-3 (PMC7494852; doi:10.1038/s41598-020-72249-3)
Supplement: Supplementary file 1 — Supplementary information. [file 41598_2020_72249_MOESM1_ESM.pdf]

Supplementary material

Effects of wound dressings containing silver on skin and immune cells

Kristina Nešporová[1], Vojtěch Pavlík[1,2], Barbora Šafránková[1], Hana Vágnerová[1], Pavel Odráška[1], Ondřej Žídek[1], Natálie Císařová[3], Svitlana Skoroplyas[4,5], Lukáš Kubala[4,5,6], Vladimír Velebný[1]

[1] Contipro a.s, Dolni Dobrouc, Czech Republic
[2] Third Faculty of Medicine, Charles University, Prague, Czech Republic
[3] Faculty of Natural Sciences, Charles University, Prague, Czech Republic
[4] Department of Free Radical Pathophysiology, Institute of Biophysics, Academy of Sciences of the Czech Republic, Brno, Czech Republic
[5]Department of Experimental Biology, Faculty of Science, Masaryk University, Kotlářská 2, Brno, 611 37, Czech Republic
[6] International Clinical Research Center, St. Anne's University Hospital, Brno, Czech Republic

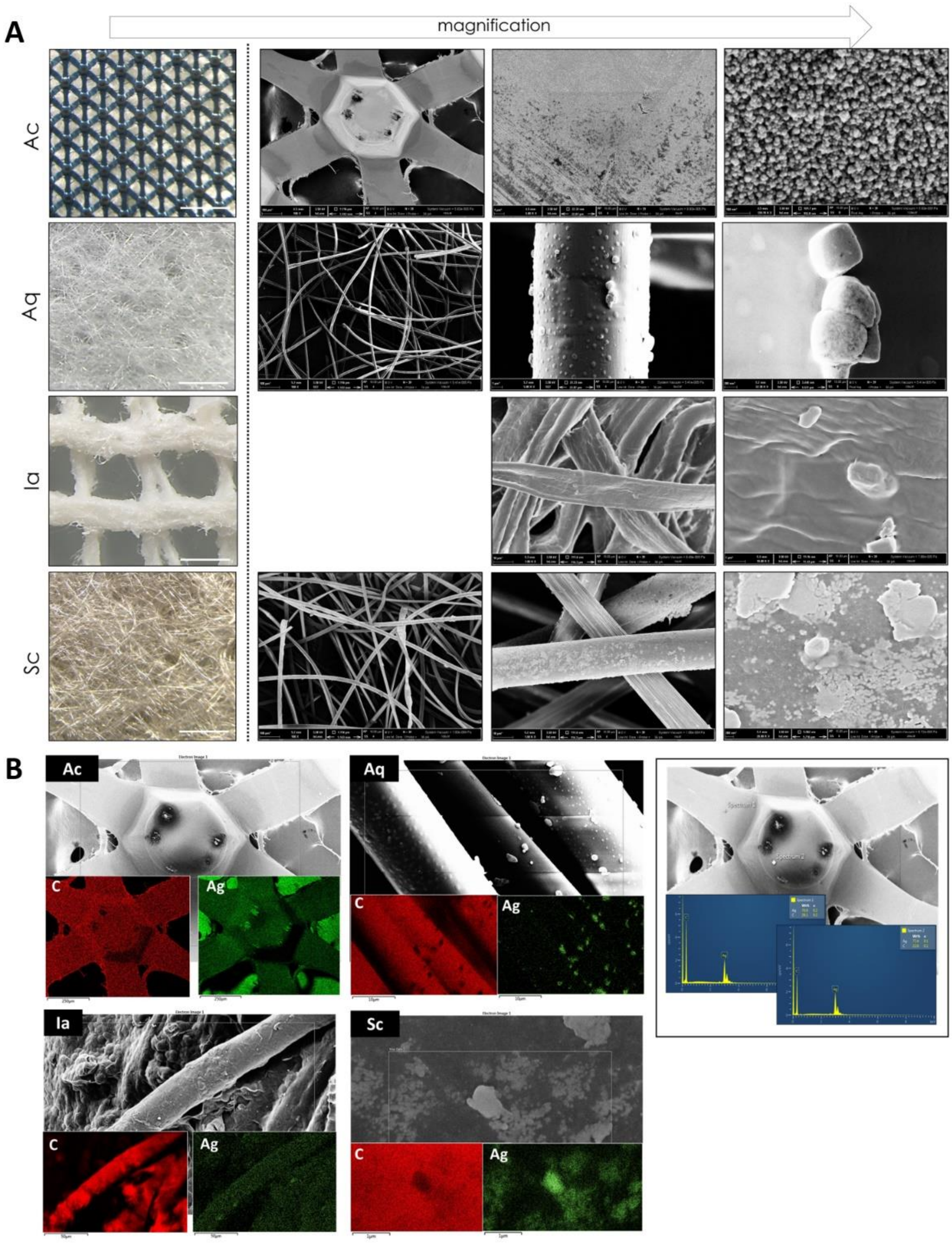

Supplementary Figure 1. (A) Macroscopic and SEM analyses of the tested dressings and **(B)** an EDX analysis of carbon and silver distributions. **(A)** The left column shows macroscopic observations of dressing structures documented by a stereomicroscope (Leica), scale bar = 1 mm. The remaining images were obtained with an electron microscope; the magnification of the pictures increases from left to right. **(B)** An EDX analysis of the dressings – grey background images represent an overview of the samples, from which fields for the analysis of carbon (C; red) or silver (Ag; green) were selected. The image in the black frame on the right represents an EDX quantification of C and Ag in Ac.

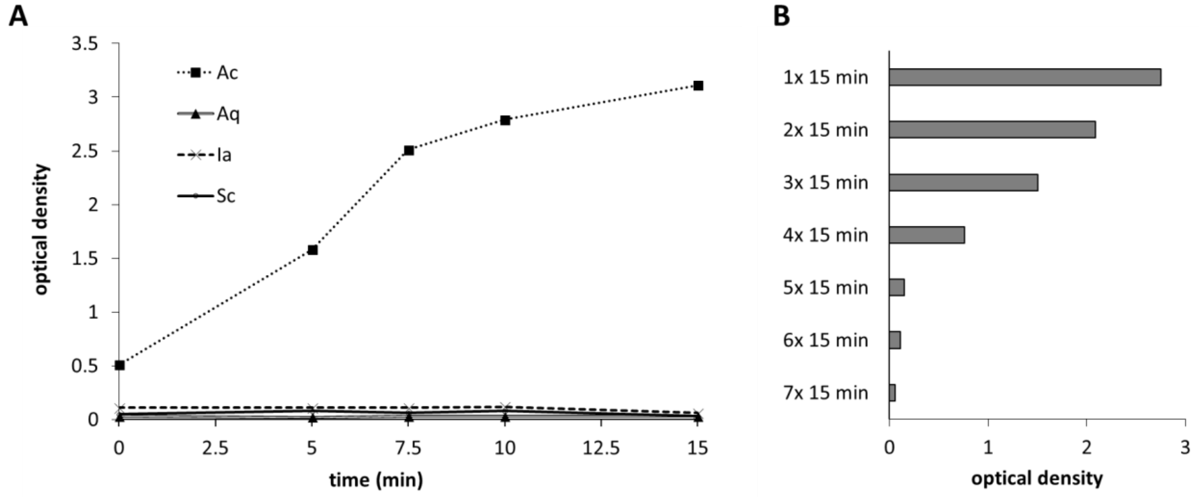

Supplementary Figure 2. The generation of hydroxyl radicals and peroxide by dressings. (A) comparison of Ac, Aq, Sc and Ia in their ability to generate oxidative species detected by the production of coloured product from TMB by HRP. (B) The capacity of Ac to generate oxidative species measured by TMB/HRP assay. The Y-axis represents the number of repeated reactions with 1 Ac sample transferred every 15 min to a new reaction tube.

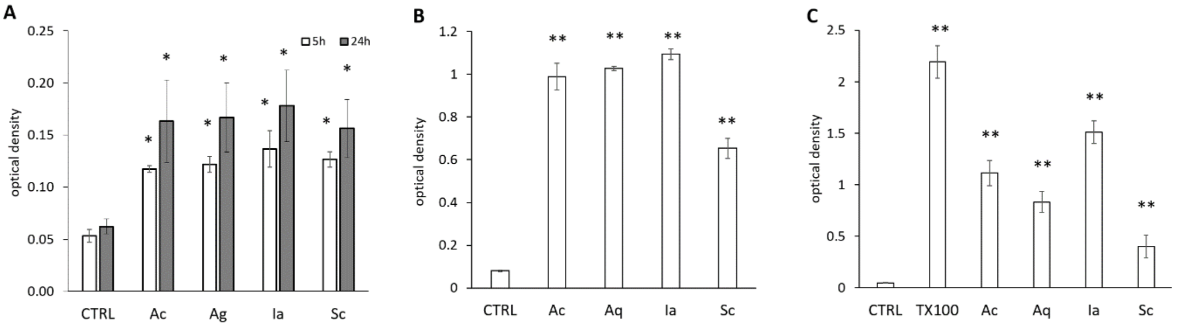

Supplementary Figure 3. LDH leakage and haemolysis after incubation of cells with silver-containing dressings. (A) LDH activity was measured in samples of isolated neutrophils after 5 and 24 h of incubation with the dressings. (B) LDH levels in whole blood after 16 h of incubation with the dressings. Results are represented as means of a minimum of three independent experiments +/- SD, * $p < 0.05$, ** $p < 0.01$. **(C)** Haemolysis in dressing-treated whole blood measured using the spectrophotometric detection of released haemoglobin. Triton X 100 was used as a positive control. Results represent means of three independent experiments +/- SD, ** $p < 0.01$ compared to the untreated control (CTRL).

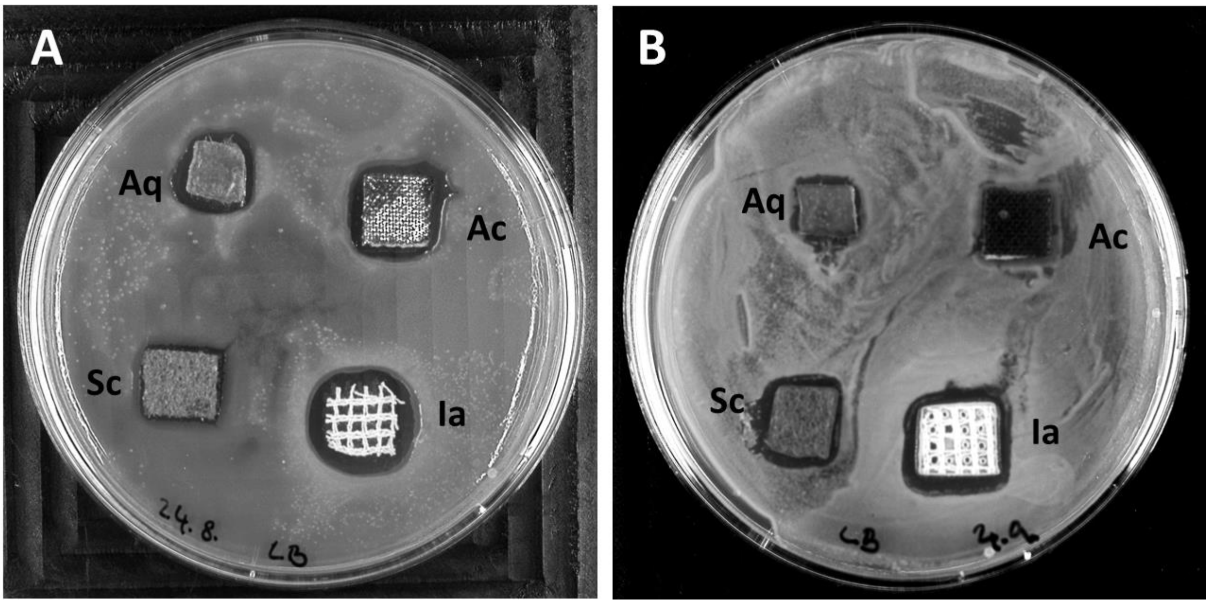

Supplementary Figure 4. Agar diffusion test of the antibacterial activity of the tested dressings against **(A)** *S. aureus* and **(B)** *P. aeruginosa* cultivated with a 1 cm^2 piece of dressing (hydrated with 10% FBS growth medium) for 24 h. Representative pictures of three independent experiments.

Supplementary Table 1. Overview of the tested wound dressings.

**Dressing name (manufacturer)**	**Composition**	**Ag form and content as claimed by the manufacturer (mg/1 cm^2)**
Acticoat^{TM} (Smith&Nephew, UK)	absorbent rayon/polyester inner core sandwiched between two outer layers of low adherent polyethylene net coated with nanocrystalline silver	nanocrystalline silver, Ag^0<br>not reported
Aquacel® Ag Hydrofiber® (Convatec, UK)	non-woven textile composed of sodium carboxymethylcellulose (CMC) and ionic silver	ionic silver (AgCl)<br>1.2% Ag^+ (0.12 mg of Ag^+/ cm^2)
Ialugen Plus® (IBI, Czech Republic)	dressing impregnated with cream containing sodium hyaluronate, silver sulfadiazine, macrogol 4000, 85% glycerol, water	silver sulfadiazine (SSD)<br>0.4 mg of SSD / cm^2 (0.12 mg of Ag^+ / cm^2)
Silvercel® Hydro-Alginate (Systagenix, UK)	non-woven pad composed of high G (guluronic acid) alginate, CMC and silver coated fibers	ionic silver<br>not reported

Supplementary Table 2. Minimal inhibitory concentrations (MIC) of silver contained in the tested dressings. The dressings were incubated for three days in DMEM medium with 10 % FBS. MIC was assessed by the dilution method. The table shows dilutions of extracts that were efficient in preventing the growth of bacteria, and the corresponding calculated final amounts of silver in the samples at the minimal inhibitory concentrations.

Dressing	MIC dilution (µg of Ag/mL)	
	S. aureus	*P. aeruginosa*
Aq	- (>18.1 ± 5.6)	1× (18.1 ± 5.6)
Ac	1× (21.8 ± 4.4)	2× (10.9 ± 2.2)
Sc	- (>3.5 ± 0.4)	1× (3.5 ± 0.4)
Ia	1× (45.9 ± 23.0)	2× (22.95 ± 11.5)
